# Development of a nomogram prediction model for gait speed trajectories in persons with knee osteoarthritis

**DOI:** 10.1038/s41598-023-37193-y

**Published:** 2023-07-12

**Authors:** Peiyuan Liu, Cui Wang, Hongbo Chen, Shaomei Shang

**Affiliations:** 1 School of Nursing, Peking University, 38 Xueyuan Road, Haidian District, Beijing, 100191 China; 2 School of Public Health, Peking University, 38 Xueyuan Road, Haidian District, Beijing, 100191 China

**Keywords:** Osteoarthritis, Risk factors

## Abstract

To examine heterogeneous trajectories of 8-year gait speed among patients with symptomatic knee osteoarthritis (KOA) and to develop a nomogram prediction model. We analyzed data from the Osteoarthritis Initiative (OAI) assessed at baseline and follow-up over 8 years (n = 1289). Gait speed was measured by the 20-m walk test. The gait speed trajectories among patients with KOA were explored by latent class growth analysis. A nomogram prediction model was created based on multivariable logistic regression. Three gait speed trajectories were identified: the fast gait speed group (30.4%), moderate gait speed group (50.5%) and slow gait speed group (19.1%). Age ≥ 60 years, female, non-white, nonmarried, annual income < $50,000, obesity, depressive symptoms, comorbidity and WOMAC pain score ≥ 5 were risk factors for the slow gait trajectory. The area under the ROC curve of the prediction model was 0.775 (95% *CI* 0.742–0.808). In the external validation cohort, the AUC was 0.773 (95% *CI* 0.697–0.848). Heterogeneous trajectories existed in the gait speed of patients with KOA and could be predicted by multiple factors. Risk factors should be earlier identified, and targeted intervention should be carried out to improve physical function of KOA patients.

## Introduction

Knee osteoarthritis (KOA) is a chronic and degenerative disease that causes joint pain, stiffness, and functional limitations. There is evidence that knee OA contributes to global disability^1^ and loss of function^2^. It is estimated that there were over 100 million symptomatic knee OA patients in China^3^ and 9.3 million in the United States^4^. KOA is associated with a decrease in quality of life^5^, leading to increased economic and humanistic burdens^2^.Gait speed (GS) is an important indicator of functional state and health condition. Generally, 20-m walk is used as a standard outcome measure of functional performance for KOA ^6^, which is a predictor for diseases and death ^7,8^. Recent studies have shown that many factors can affect gait speed, mainly including sociodemographic variables such as age, gender and race^9,10^, as well as health-related variables like BMI, comorbidity, pain and depression^9–12^.To date, it has been demonstrated that there are distinct disease trajectories of function^13^, quality of life^14^, knee pain^15^ and structural progression^16^, suggesting that KOA patients have interindividual variability. A previous study has described gait speed trajectories in participants with or at risk of KOA^17^. However, it remains unclear how trajectories of gait speed change over time in KOA patients, nor predicting factors of trajectories. Understanding the heterogeneity in the longitudinal gait speed trajectories is of importance to clarify the natural history. Recognizing the predicting factors is helpful to find individuals at risk and set specific intervention strategies to improve physical function. Therefore, the purpose of this study was to explore gait speed trajectories of people with KOA, and to develop a nomogram predicting model to identify individuals with low gait speed.

## Methods

### Study population

Participants included in the study were collected form the Osteoarthritis Initiative (OAI), an ongoing, multicenter, prospective observational, longitudinal cohort of the risk factors and natural history of OA, including 4796 participants between 45 and 79 years. Detailed information of rationale and approach for the OAI can be found at https://data-archive.nimh.nih.gov/oai/. Data used in this study includes the baseline visit and eight years of follow-up for analyses. An index knee for each participant was set based on the Kellgren–Lawrence (KL) grade at baseline. The knee with higher KL grade was set as the index knee. In case an equal KL grade in both knees, the knee with a higher Western Ontario and McMaster Osteoarthritis Index (WOMAC) pain score was set as the index knee. The index knee was randomly set if knees had the same KL grade and WOMAC pain score. Participants with KOA in at least one knee at baseline were enrolled. KOA was defined as an index knee with KL grade ≥ 2 and having pain, aching, or stiffness in or around the index knee in most days for at least 1 month during the past 12 months. Individuals without 20-m walk gait speed information at baseline or all the follow-up visits were excluded. Supplementary Fig. 1 shows the flow diagram of this study. The OAI study was approved by the institutional review boards at all OAI clinical centers, the coordinating center (Memorial Hospital of Rhode Island Institutional Review Board, The Ohio State University’s Biomedical Sciences Institutional Review Board, University of Pittsburgh Institutional Review Board, University of Maryland Baltimore-Institutional Review Board, and Committee on Human Research at University of California, San Francisco; approval number 10-00532), the NIH, OAI investigators and private funding partners. All methods were performed in accordance the relevant guidelines and regulations. Informed consent was taken from subjects before the study so no additional approval was needed.

### Gait speed

Gait speed was measured by a 20-m walk test, which was used frequently in KOA studies and had high sensitivity and test–retest reliability^6^. Participants were instructed to walk at a comfortable pace and allowed to use walking aids. The stopwatch was starting when participants began walking and was stopped as soon as participants stepping over the finishing line. Each participant performed 2 20-m walk trials and gait speed was calculated using the average of 2 walking gait speeds. Gait speed at baseline and 1/2/3/4/6/8 year (s) follow-up visits were collected.

### Factors associated with gait speed

Based on previous studies^10,18–25^, potential factors influencing gait speed of KOA were included. The potential factors were as follows: baseline age, gender (men, women), race (white or Caucasian, other), education (tertiary, secondary, none or primary), marital status (married, other), income (≥ 50,000$, < 50,000$), smoking history (yes, no), drinking history (yes, no), history of knee injury that limited walking ability for at least 2 days (yes, no), history of knee surgery (yes, no) and comorbidity (yes, no) were measured by self-report.

Radiographic knee osteoarthritis (ROA) was assessed from weight-bearing posteroanterior and lateral fixed flexion radiographic evaluations of both knees^26^. Radiographs were independently graded twice among three expert readers (two rheumatologists and a musculoskeletal radiologist) for joint space narrowing and osteophytes in the tibiofemoral joint according to KL criteria (grades 0–4) ^27^.

Modifiable factors included knee pain, obesity and depressive symptoms. Knee pain was evaluated by the WOMAC pain subscale^28^, which was scored from 0 to 20, and higher scores indicated higher pain severity. Obesity was classified as a BMI ≥ 30 kg/m^2^. Depressive symptoms were defined as the Center for Epidemiological Studies Depression (CES-D) score ≥ 16^29^.

### Statistical analyses

Data were analyzed using Mplus version 7.0, STATA version 15.0 and R software 4.2.2. Baseline characteristics of participants were expressed as frequencies or percentages and compared by performing chi-square tests for categorical variables. Restricted cubic spline models were used to describe the possible nonlinear associations between continuous variables and gait speed. Missing data were imputed with the median and the mode in STATA.

Trajectories of gait speed were identified by using latent class growth analysis (LCGA) in Mplus, which is a type of mixture modeling to identify possible distinct subgroups. The optimal number of trajectory groups was determined by considering the smallest Akaike information criterion (AIC), Bayesian information criterion (BIC) and sample size adjusted BIC (aBIC), entropy values closest to 1, bootstrap likelihood ratio test (BLRT) *P*-value < 0.05 which means the model with n classes is better than the model with n-1 classes, and the number of members per class > 1% of the total cohort. The Guidelines for Reporting on Latent Trajectory Studies (GRoLTS)-Checklist was used for the reporting of the trajectory analysis^30^ (Supplementary Table 1). Missing data for gait speed were handled under missing at random and no imputation of gait speed was undertaken.

To make full use of the data, the enrolled participants were randomly allocated to the training cohort (80%, n = 1039) and validation cohort (20%, n = 250). The univariate and least absolute shrinkage and selection operator (LASSO) model was used to determine risk factors. Multivariate logistic regression analysis was carried out to construct the nomogram prediction model. The area under the receiver operating characteristic curve (AUC) was used to evaluate the predictive capacity of the prediction model. The calibration curve and Hosmer–Lemeshow goodness-of-fit test were performed to analyze the calibration of the nomogram. The model development and reporting were in accordance with the Transparent Reporting of a Multivariable Prediction Model for Individual Prognosis or Diagnosis (TRIPOD) checklist^31^. A two-tailed *P*-value < 0.05 was considered statistically significant.

### Ethics approval and consent to participate

The OAI study was approved by the institutional review boards at all OAI clinical centers, the coordinating center (University of California, San Francisco, USA; approval number 10-00532), the NIH, OAI investigators and private funding partners. Informed consent was taken from subjects before the study so no additional approval was needed.

## Results

### Baseline characteristics

A total of 1289 patients were included in the study, including 709 patients aged 60 years or older at baseline. There were 562 males and 727 females; 908 were white and 381 were black/yellow/other races; 824 were married and 465 were single/divorced/widowed. The baseline characteristics of the training cohort and validation cohort are listed in Table 1, and there was no significant difference in variables between the two cohorts (*P* > 0.05).

**Table 1:** Baseline characteristics of the training and validation cohorts.

Characteristic	All patients (n = 1289)	Training cohort (n = 1039)	Validation cohort (n = 250)	*P* value
Age, year				0.724
< 60	580 (45.00)	470 (45.24)	110 (44.00)	
≥ 60	709 (55.00)	569 (54.76)	140 (56.00)	
Gender				1.000
Female	727 (56.40)	586 (56.40)	141 (56.40)	
Male	562 (43.60)	453 (43.60)	109 (43.60)	
Race				0.655
Other	381 (29.56)	310 (29.84)	71 (28.40)	
White or Caucasian	908 (70.44)	729 (70.16)	179 (71.60)	
Marital status				0.447
Other	465 (36.07)	380 (36.57)	85 (34.00)	
Married	824 (63.93)	659 (63.43)	165 (66.00)	
Education				0.422
None/primary	260 (20.17)	209 (20.11)	51 (20.40)	
Secondary	611 (47.40)	501 (48.22)	110 (44.00)	
Tertiary	418 (32.43)	329 (31.67)	89 (35.60)	
Income, $				0.114
< 50,000	547 (42.44)	452 (43.50)	95 (38.00)	
≥ 50,000	742 (57.56)	587 (56.50)	155 (62.00)	
Smoking history				0.829
No	575 (44.61)	465 (44.75)	110 (44.00)	
Yes	714 (55.39)	574 (55.25)	140 (56.00)	
Drinking history				0.517
No	776 (60.20)	630 (60.64)	146 (58.40)	
Yes	513 (39.80)	409 (39.36)	104 (41.60)	
Obesity				0.555
No	659 (51.12)	527 (50.72)	132 (52.80)	
Yes	630 (48.88)	512 (49.28)	118 (47.20)	
Depressive symptoms				0.208
No	1113 (86.35)	891 (85.76)	222 (88.80)	
Yes	176 (13.65)	148 (14.24)	28 (11.20)	
Comorbidity				0.727
No	919 (71.30)	743 (71.51)	176 (70.40)	
Yes	370 (28.70)	296 (28.49)	74 (29.60)	
History of knee injury				0.190
No	1209 (93.79)	979 (94.23)	230 (92.00)	
Yes	80 (6.21)	60 (5.77)	20 (8.00)	
History of knee surgery				0.471
No	815 (63.23)	652 (62.75)	163 (65.20)	
Yes	474 (36.77)	387 (37.25)	87 (34.80)	
KLG				0.708
2	585 (45.38)	472 (45.43)	113(45.20)	
3	504 (39.10)	402 (38.69)	102 (40.80)	
4	200 (15.52)	165 (15.88)	35 (14.00)	
WOMAC pain				0.973
< 5	589 (45.69)	475 (45.72)	114 (45.60)	
≥ 5	700 (54.31)	564 (54.28)	136 (54.40)	

Obesity was defined as BMI ≥ 30 kg/m^2^, depressive symptoms were defined as CES-D (catchment-area epidemiology survey-depression) score ≥ 16.

*KLG* Kellgren–Lawrence grade, *WOMAC *Western Ontario & McMaster Universities Osteoarthritis Index.

### Trajectories

**Figure 1:**
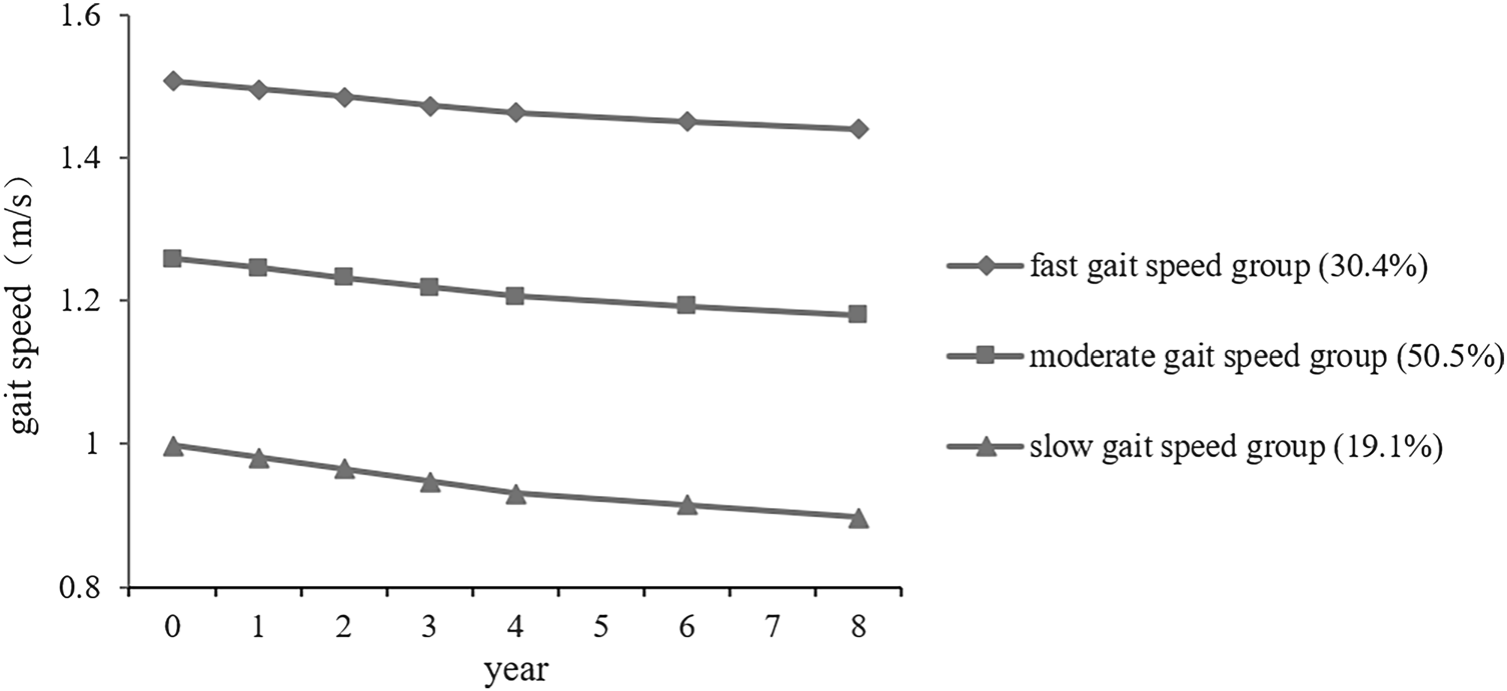
Three distinct trajectories of gait speed change over 8 years based on the 3-class model.

The 20-m walk gait speeds at baseline and 6 follow-up visits were 1.27 ± 0.23, 1.28 ± 0.23, 1.28 ± 0.22, 1.26 ± 0.22, 1.25 ± 0.22, 1.24 ± 0.24 and 1.20 ± 0.23 m/s, respectively. The LCGA model was used to fit the heterogeneous trajectories of the 20-m gait speed, and the results are shown in Table 2. According to the model fit results, it was considered most appropriate to divide the 20-m gait speed into three categories, named the fast gait speed group (n = 392), the moderate gait speed group (n = 651) and the slow gait speed group (n = 246) according to their variation characteristics (Fig. 1). Approximately 19.1% of patients were in the slow gait speed group with a gait speed decrease of 0.100 m/s or 10.06% over 8 years. The gait speed of the fast and moderate gait speed group underwent a decrease of 0.081 m/s (6.39%) and 0.068 m/s (4.48%), respectively.

**Table 2:** Model Fit Comparison for Latent Class Growth Analysis (LCGA).

Classes	AIC	BIC	aBIC	Entropy	BLRT	Percentage of individuals in class			
1	−1002.120	−955.666	−984.254	–	–	1			
2	−4504.117	−4442.177	−4480.295	0.839	< 0.001	0.491	0.509		
3	−6256.066	−6178.642	−6226.290	0.883	< 0.001	0.191	0.505	0.304	
4	−7041.393	−6948.484	−7005.661	0.877	< 0.001	0.332	0.437	0.080	0.151

### Univariate analysis of trajectories

The fast and moderate gait speed group were combined into the good gait speed group. Univariate analysis showed that there existed significant differences in age, gender, race, marital status, education level, annual income, drinking history, obesity, depressive symptoms, comorbidity and pain between the good gait speed group and the slow gait speed group (*P* < 0.05) (Supplementary Table 2).

### Nomogram construction

**Figure 2:**
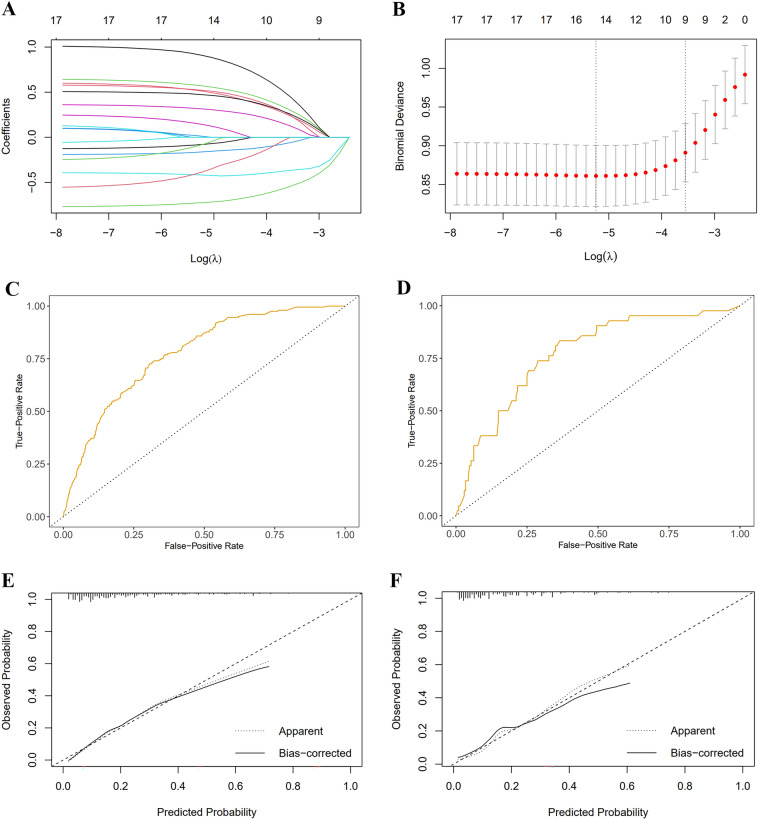
Nomogram construction and validation. (**A**) Lasso coefficient profiles of the 9 predictors. (**B**) Optimal predictor (lambda) selection in the Lasso model with fivefold cross validation by minimum criteria. (**C**) Receiver operating characteristic curve analysis in the training cohorts. (**D**) Receiver operating characteristic curve analysis in the validation cohorts. (**E**) Calibration curve analysis in the training cohorts. (**F**) Calibration curve analysis in the validation cohorts.

The baseline variables were screened by the LASSO regression model, and the best value of parameter λ was screened through cross-validation. The selected predictors were 9 when the overall deviation of the model was the minimum. Age, gender, race, marital status, annual income, obesity, comorbidity, depressive status, and pain were independent predictors of slow gait speed trajectory (Fig. 2).

**Figure 3:**
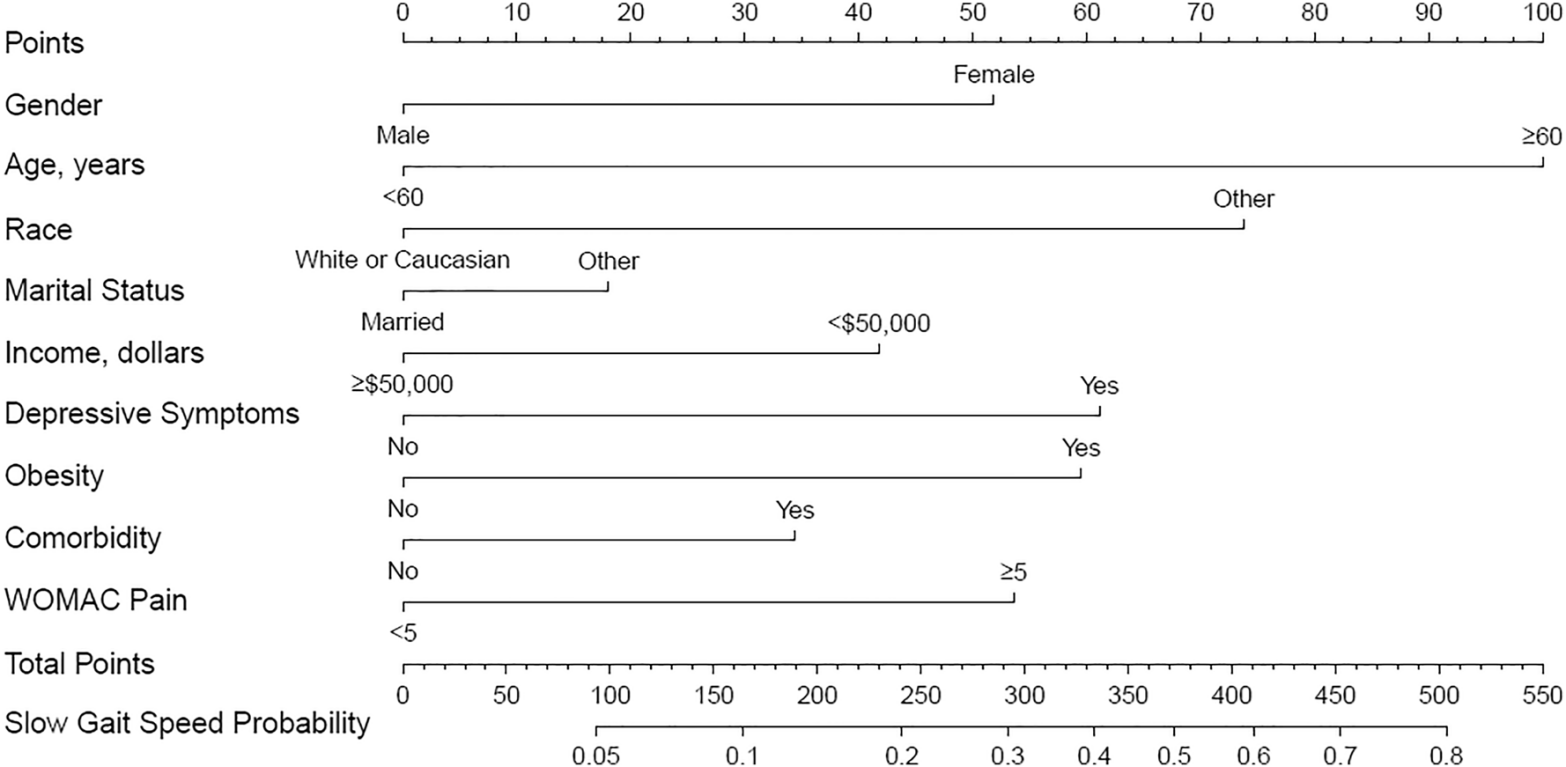
Nomogram of prediction model.

The above 9 factors were used to construct the nomogram prediction model using logistic regression, with the walking speed trajectory (0 = good gait speed trajectory, 1 = slow gait speed trajectory) as the dependent variable (Fig. 3). The results of parameter estimation and regression are shown in Table 3.

**Table 3:** Univariate and multivariate logistic regression analyses of training cohort.

variables	Univariate model			Multivariable model		
	*OR*	95%*CI*	*P* value	*OR*	95%*CI*	*P* value
Age, year						
< 60						
≥ 60	2.188	1.577–3.037	< 0.001	2.873	1.983–4.215	< 0.001
Gender						
Female						
Male	0.405	0.289–0.568	< 0.001	0.578	0.397–0.835	0.004
Race						
Other						
White or Caucasian	0.323	0.236–0.443	< 0.001	0.459	0.319–0.660	< 0.001
Marital status						
Other						
Married	0.419	0.307–0.571	< 0.001	0.827	0.570–1.204	0.318
Education						
None/primary						
Secondary	0.738	0.541–1.005	0.052			
Tertiary	0.647	0.456–0.918	0.012			
Income, $						
< 50,000						
≥ 50,000	0.330	0.240–0.455	< 0.001	0.644	0.443–0.933	0.020
Smoking history						
No						
Yes	1.140	0.837–1.554	0.404			
Drinking history						
No						
Yes	0.532	0.381–0.744	< 0.001			
Obesity						
No						
Yes	2.377	1.725–3.275	< 0.001	1.874	1.320–2.674	< 0.001
Depressive symptoms						
No						
Yes	2.442	1.666–3.579	< 0.001	1.906	1.220–2.959	0.004
Comorbidity						
No						
Yes	2.112	1.535–2.906	< 0.001	1.438	1.008–2.043	0.044
History of knee injury						
No						
Yes	1.264	0.681–2.347	0.466			
History of knee surgery						
No						
Yes	0.725	0.523–1.005	0.050			
KLG						
2						
3	1.259	0.923–1.717	0.147			
4	0.982	0.645–1.495	0.932			
WOMAC pain						
< 5						
≥ 5	2.511	1.780–3.504	< 0.001	1.761	1.227–2.546	0.002

Obesity was defined as BMI ≥ 30 kg/m^2^, depressive symptoms were defined as CES-D (catchment-area epidemiology survey-depression) score ≥ 16.

*KLG* Kellgren–Lawrence grade, *WOMAC* Western Ontario & McMaster Universities Osteoarthritis Index.

### Nomogram validation

The AUC of the nomogram was 0.775 (95% *CI* 0.742–0.808) in the training cohort and 0.773 (95% *CI* 0.697–0.848) in the validation cohort, indicating high accuracy in predicting the risk of the slow gait speed trajectory (Fig. 2).

Calibration curves were employed to evaluate the calibration (Fig. 2), demonstrating the high degree of agreement between the predicted and observed probabilities. The Hosmer–Lemeshow goodness-of-fit test indicated that the nomogram had a good fit for the data (${\chi }^{2}$ = 10.64, *P* = 0.223).

Obesity was defined as BMI ≥ 30 kg/m^2^, Depressive symptoms were defined as CES-D (catchment-area epidemiology survey-depression) score ≥ 16, KLG Kellgren–Lawrence grade, WOMAC Western Ontario & McMaster Universities Osteoarthritis Index.

## Discussion

In this study, we used LCGA to identify distinct trajectories of gait speed over 8 years and developed and internally validated a nomogram prediction model to identify patients with KOA following the slow gait speed trajectory. We found that the gait speed remained slowly decreased in all three trajectories, and there existed a minority with low-level gait speed that had not reached 1.0 m/s at baseline and all follow-up visits. Due to our concern about the worst gait speed trajectory, we combined the high-level and medium-level trajectories into one trajectory named good gait speed trajectory. The nomogram prediction model included nine easily obtained risk factors, including age, gender, race, marital status, annual income, obesity, comorbidity, depressive status, and pain. To our knowledge, this is the first study to use gait speed detected by the 20-m walk test to identify distinct trajectories in patients with KOA. Additionally, we used baseline characteristics to characterize patients in different trajectories and develop a nomogram model to predict the progression of gait speed, which was suggested by the results to be used to aid the early intervention of KOA.

In another study, Daniel et al. found 5 distinct trajectories in patients with or at the risk of KOA over 4 years using group-based trajectory modeling, one of them (5%) with a relatively rapid decrease in gait speed^17^. However, in this study we identified that 19.1% of patients with KOA were in the slow gait speed trajectory with a decrease of 10.06% over 8 years. Compared with those in the good gait speed trajectory, patients with KOA in the slow gait speed trajectory were mainly characterized by old age, female, low annual income, comorbidity, obesity, depressive symptoms, and high-level pain. For patients with KOA, maintaining a certain physical activity ability is crucial to independent living. Many daily life circumstances require patients' gait speed. For example, the minimum gait speed of 1.22 m/s is required to safely cross a street with many timed pedestrian traffic lights^32^. In addition, the 2019 Asian Working Group for Sarcopenia defined low physical performance as gait speed less than 1.0 m/s^33^, while the gait speed of KOA patients in the slow gait speed trajectory could not reach this standard at baseline and all the follow-up visits, which suggests that early targeted intervention should be carried out to improve their physical mobility.

The heterogeneity trajectories of gait speed decreased slowly over time. Gilbert et al. proposed that for patients with KOA, the important gait speed difference based on the 20-m walk test was between 0.068 and 0.115 m/s^34^, and patients in the slow gait speed trajectory reached this threshold during the 8-year follow-up visits. Results suggested that the gait speed of patients with KOA in the slow gait speed trajectory did deteriorate significantly during the follow-up visits, and the gait speed trajectory of KOA patients should be identified early.

Few studies have previously taken concern of prediction model development for slow gait speed in patients at high risk of KOA^9^ and gait speed change after education and exercise therapy in patients with KOA^35^. In contrast to the above studies, we first identified distinct trajectories of progression with similar gait speed changes and developed a prediction model to recognize patients belonging to the slow gait speed trajectory, providing new insights into understanding gait speed.

A variety of unmodifiable risk factors for gait speed have been discussed, which is similar to findings in this study, including old age^19,21,25^, female^10^, non-white^24^, low income^10^ and comorbidity^20^. Race-related differences in gait speed trajectories are possible ascribed to accumulated exposure of unmeasured factors, such as residential conditions, intergenerational wealth transferring, or adverse health events^36^, which can lead to more opportunities relevant to maintaining gait speed for whites, including accessibility and receipt of medical services^37^, and safe walking environments. Since income is inextricably associated with race in the United States, participants with higher income are also more likely to get access to potential chances of maintenance in gait speed. Although there is no clear evidence, we found that married patients with KOA had faster gait speed, which might be related to more connubial care, less perceived fatigability^38^, and motivated physical activities form spouse^39^. In general, a higher KL grade indicates worse physical function, whereas no significant correlation was found in this study between the KL grade and the slow gait speed trajectory, which might be because the disease symptoms rather than the structural progression itself were the main factor affecting gait speed in symptomatic KOA patients.

As for modifiable variables, obesity is related to low gait speed with causing cartilage decomposition and leading to degenerative changes of the knee joint and reducing physical activity function in patients with KOA^40,41^. Previous studies identified that individuals with obesity had slow gait speed^25^ and especially muscle-reducing obesity was associated with slow gait speed in KOA patients^42^. Consequently, tackling obesity in patients with KOA is of great significance to maintain or improve gait speed, and effective strategies incorporate bariatric surgeries, weight reduction diets, exercise regimens and cognitive behavioral strategies^43,44^, which can generate greater weight loss when combined together^45^. Morone et al. found that with the increase in pain level, the gait speed of KOA patients decreased significantly^23^, which was consistent with findings in this study. As the most troublesome symptom in patients with KOA, knee pain can result in limitation of activities, nevertheless, with the absence of treatments to eliminate pain. It’s common for individuals to take analgesics or receive total knee replacement surgeries on account of knee pain^46^, and there exists some conservative treatments relieving pain, including the low-calorie diet, exercise intervention and physical therapy^43^. A relationship between slow gait speed and depressive symptoms has been found in a recent study^18^, while anxiety-related pain response, rather than anxiety and depression, has been suggested as an important factor related to slow gait speed in patients with lower limb osteoarthritis^22^, indicating that the causal relationship between depressive symptoms and gait speed needs further study. Common in patients with KOA^47^, depressive symptoms are generally attenuated with participation in exercise programs^48^, drug therapy, and psychotherapy, more safer as well as acceptable, mainly including cognitive behavioral therapy and mindfulness-based interventions.

There are several important limitations in this study. First, due to the observational nature of the OAI, treatment interventions including medications and injections continued to be received and might have an impact on trajectory patterns, while our analysis did not take these factors into account. In addition, although variables included in the prediction model were significantly associated with the slow gait speed trajectory, the mechanisms of these associations need advanced research. Finally, we did not conduct external validation of the model, which is necessary before its use in clinical studies to select patients.

## Conclusion

In conclusion, this study revealed 3 distinct trajectory patterns in patients with KOA over 8 years. While a majority of patients had high-level or medium-level gait speed, those in the slow gait speed trajectory were more likely to undergo worse clinical outcomes, indicating physical function deterioration. A nomogram prediction model was developed and validated, which manifested that presence of age ≥ 60 years, female, White, married, annual income < 50,000 dollars, obesity, comorbidity, depressive symptoms, and WOMAC pain score ≥ 5 can precisely predict the slow gait speed trajectory. Our prediction model may contribute to identifying individuals at risk of the slow gait speed trajectory within 8 years in the early stage, allowing education and targeted intervention of modifiable risk factors for improving the physical function of patients with KOA.

## Supplementary Information

Supplementary Information.

## Data Availability

The datasets used and/or analyzed during the current study are available from the corresponding author on reasonable request.

## Abbreviations

KOA: Knee osteoarthritis
OAI: Osteoarthritis Initiative
ROA: Radiographic knee osteoarthritis
GS: Gait speed
BMI: Body mass index
WOMAC: Western Ontario and McMaster Universities Osteoarthritis Index
KLG: Kellgren–Lawrence grade
CES-D: Center for Epidemiologic Studies Depression Scale
LCGA: Latent class growth analysis
AIC: Akaike information criterion
BIC: Bayesian information criterion
BLRT: Bootstrap likelihood ratio test
LASSO: Least absolute shrinkage and selection operator
ROC: Receiver operating characteristic
AUC: Area under the receiver operating characteristic curve
TRIPOD: Transparent Reporting of a Multivariable Prediction Model for Individual Prognosis or Diagnosis

## References

[1] Vos T (2016). Global, regional, and national incidence, prevalence, and years lived with disability for 310 diseases and injuries, 1990–2015: A systematic analysis for the Global Burden of Disease Study 2015. Lancet.

[2] Xie F (2016). Economic and humanistic burden of osteoarthritis: A systematic review of large sample studies. Pharmacoeconomics.

[3] Tang X (2016). The prevalence of symptomatic knee osteoarthritis in China: Results from the China Health and Retirement Longitudinal Study. Arthritis Rheumatol..

[4] Lawrence RC (2008). Estimates of the prevalence of arthritis and other rheumatic conditions in the United States: Part II. Arthritis Rheum..

[5] Törmälehto S (2018). Health-related quality of life in relation to symptomatic and radiographic definitions of knee osteoarthritis: Data from Osteoarthritis Initiative (OAI) 4-year follow-up study. Health Qual. Life Outcomes..

[6] Motyl JM, Driban JB, McAdams E, Price LL, McAlindon TE (2013). Test–retest reliability and sensitivity of the 20-meter walk test among patients with knee osteoarthritis. BMC Musculoskelet. Disord..

[7] Alenazi AM (2021). Gait speed as a predictor for diabetes incidence in people with or at risk of knee osteoarthritis: A longitudinal analysis from the Osteoarthritis Initiative. Int. J. Environ. Res. Public Health.

[8] Yates T (2017). Association of walking pace and handgrip strength with all-cause, cardiovascular, and cancer mortality: A UK Biobank observational study. Eur. Heart J..

[9] Sharma L (2019). Development and validation of risk stratification trees for incident slow gait speed in persons at high risk for knee osteoarthritis. Ann. Rheum. Dis..

[10] King LK, Kendzerska T, Waugh EJ, Hawker GA (2018). Impact of osteoarthritis on difficulty walking: A population-based study. Arthritis Care Res..

[11] Yamaguchi N (2018). Pain deterioration within 1 year predicts future decline of walking ability: A 7-year prospective observational study of elderly female patients with knee osteoarthritis living in a rural district. Geriatr. Orthop. Surg. Rehabil..

[12] Bindawas S (2016). Relationship between frequent knee pain, obesity, and gait speed in older adults: Data from the Osteoarthritis Initiative. Clin. Interv. Aging..

[13] White DK, Neogi T, Nguyen UDT, Niu J, Zhang Y (2016). Trajectories of functional decline in knee osteoarthritis: The Osteoarthritis Initiative. Rheumatology.

[14] Törmälehto S (2019). Eight-year trajectories of changes in health-related quality of life in knee osteoarthritis: Data from the Osteoarthritis Initiative (OAI). PLoS ONE.

[15] Bastick AN (2015). Defining knee pain trajectories in early symptomatic knee osteoarthritis in primary care: 5-year results from a nationwide prospective cohort study (CHECK). Br. J. Gen. Pract..

[16] Deveza LA (2019). Trajectories of femorotibial cartilage thickness among persons with or at risk of knee osteoarthritis: Development of a prediction model to identify progressors. Osteoarthr. Cartil..

[17] White DK, Niu J, Zhang Y (2013). Is symptomatic knee osteoarthritis a risk factor for a trajectory of fast decline in gait speed? Results from a longitudinal cohort study. Arthritis Care Res..

[18] Poon CL (2022). Associations of the modified STarT back tool and Hospital Anxiety and Depression Scale (HADS) with gait speed and knee pain in knee osteoarthritis: A retrospective cohort study. Disabil. Rehabil..

[19] Marcum ZA (2014). Correlates of gait speed in advanced knee osteoarthritis. Pain Med..

[20] Lee SW (2020). Effect of comorbid chronic low back pain on patient-reported outcome and gait parameters in patients with symptomatic knee osteoarthritis. Am. J. Phys. Med. Rehabil..

[21] Duffell LD, Jordan SJ, Cobb JP, McGregor AH (2017). Gait adaptations with aging in healthy participants and people with knee-joint osteoarthritis. Gait Posture..

[22] Hayashi K (2016). Gait speeds associated with anxiety responses to pain in osteoarthritis patients. Pain Med..

[23] Morone NE, Abebe KZ, Morrow LA, Weiner DK (2014). Pain and decreased cognitive function negatively impact physical functioning in older adults with knee osteoarthritis. Pain Med..

[24] Sims EL (2009). Racial differences in gait mechanics associated with knee osteoarthritis. Aging Clin. Exp. Res..

[25] Kinoshita K (2021). The effect of age on the association between daily gait speed and abdominal obesity in Japanese adults. Sci. Rep..

[26] Peterfy C (2003). Comparison of fixed-flexion positioning with fluoroscopic semi-flexed positioning for quantifying radiographic joint-space width in the knee: Test–retest reproducibility. Skelet. Radiol..

[27] Kellgren JH, Lawrence JS (1957). Radiological assessment of osteo-arthrosis. Ann. Rheum. Dis..

[28] Bellamy N, Buchanan WW, Goldsmith CH, Campbell J, Stitt LW (1988). Validation study of WOMAC: A health status instrument for measuring clinically important patient relevant outcomes to antirheumatic drug therapy in patients with osteoarthritis of the hip or knee. J. Rheumatol..

[29] Smarr KL, Keefer AL (2011). Measures of depression and depressive symptoms: Beck Depression Inventory-II (BDI-II), Center for Epidemiologic Studies Depression Scale (CES-D), Geriatric Depression Scale (GDS), Hospital Anxiety and Depression Scale (HADS), and Patient Health Questionna. Arthritis Care Res..

[30] van de Schoot R, Sijbrandij M, Winter SD, Depaoli S, Vermunt JK (2017). The GRoLTS-Checklist: Guidelines for reporting on latent trajectory studies. Struct. Equ. Model. Multidiscip. J..

[31] Collins GS, Reitsma JB, Altman DG, Moons KGM (2015). Transparent reporting of a multivariable prediction model for individual prognosis or diagnosis (TRIPOD): The TRIPOD statement. BMJ-Br. Med. J..

[32] Dunlop DD, Song J, Semanik PA, Sharma L, Chang RW (2011). Physical activity levels and functional performance in the osteoarthritis initiative: A graded relationship. Arthritis Rheum..

[33] Chen L (2020). Asian Working Group for Sarcopenia: 2019 consensus update on sarcopenia diagnosis and treatment. J. Am. Med. Dir. Assoc..

[34] Gilbert AL, Song J, Cella D, Chang RW, Dunlop DD (2021). What is an important difference in gait speed in adults with knee osteoarthritis?. Arthritis Care Res..

[35] Baumbach L, List M, Grønne DT, Skou ST, Roos EM (2020). Individualized predictions of changes in knee pain, quality of life and walking speed following patient education and exercise therapy in patients with knee osteoarthritis—A prognostic model study. Osteoarthr. Cartil..

[36] Thorpe RJ (2011). Race, socioeconomic resources, and late-life mobility and decline: Findings from the Health, Aging, and Body Composition Study. J. Gerontol. A Biol. Sci. Med. Sci..

[37] Bowen ME, Gonzalez HM (2008). Racial/ethnic differences in the relationship between the use of health care services and functional disability: The health and retirement study (1992–2004). Gerontologist..

[38] Moored KD (2022). Prospective associations between physical activity and perceived fatigability in older men: Differences by activity type and baseline marital status. J. Gerontol. Ser. A.

[39] Pettee KK (2006). Influence of marital status on physical activity levels among older adults. Med. Sci. Sports Exerc..

[40] Kulkarni K, Karssiens T, Kumar V, Pandit H (2016). Obesity and osteoarthritis. Maturitas.

[41] Raud B (2020). Level of obesity is directly associated with the clinical and functional consequences of knee osteoarthritis. Sci. Rep..

[42] Godziuk K, Prado CM, Woodhouse LJ, Forhan M (2019). Prevalence of sarcopenic obesity in adults with end-stage knee osteoarthritis. Osteoarthr. Cartil..

[43] Panunzi S (2021). Comparative efficacy of different weight loss treatments on knee osteoarthritis: A network meta-analysis. Obes. Rev..

[44] Wluka AE, Lombard CB, Cicuttini FM (2013). Tackling obesity in knee osteoarthritis. Nat. Rev. Rheumatol..

[45] Bales CW, Porter Starr KN (2018). Obesity interventions for older adults: diet as a determinant of physical function. Adv. Nutr..

[46] Hawker GA (2000). Differences between men and women in the rate of use of hip and knee arthroplasty. N. Engl. J. Med..

[47] Stubbs B, Aluko Y, Myint PK, Smith TO (2016). Prevalence of depressive symptoms and anxiety in osteoarthritis: a systematic review and meta-analysis. Age Ageing..

[48] Hurley M (2018). Exercise interventions and patient beliefs for people with hip, knee or hip and knee osteoarthritis: A mixed methods review. Cochrane Database Syst. Rev..

