# Supplementary material for: Development of a nomogram prediction model for gait speed trajectories in persons with knee osteoarthritis
Source: Sci Rep. 2023 Jul 12;13:11291. doi: 10.1038/s41598-023-37193-y (PMC10338457; doi:10.1038/s41598-023-37193-y)
Supplement: Supplementary file 1 — Supplementary Information. [file 41598_2023_37193_MOESM1_ESM.docx]

Supplementary Table 1. Final List of Items of the Guidelines for Reporting on Latent Trajectory Studies (GRoLTS) Checklist: Guidelines for Reporting on Latent Trajectory Studies

| Checklist Item Reported? | Yes/No | Comment |
| --- | --- | --- |
| 1. Is the metric of time used in the statistical model reported? | Y |  |
| 2. Is information presented about the mean and variance of time within a wave? | N | The mean (SD) number of days from baseline to the 1-year (n=4,496), 2-year (n=4,324), 3-year (n=4,270), 4-year (n=4,256), 6-year (n=3,823) and 8-year (n=3,953) visits were available for the whole OAI cohort and were 386.35 (43.33), 745.38 (37.62), 1108.80 (37.12), 1470.81 (36.42), 2195.96 (44.18), 2925.49 (75.27), respectively. |
| 3a. Is the missing data mechanism reported?  3b. Is a description provided of what variables are related to attrition/missing data?  3c. Is a description provided of how missing data in the analyses were dealt with? | Y  N  Y |  |
| 4. Is information about the distribution of the observed variables included? | Y |  |
| 5. Is the software mentioned? | Y |  |
| 6a. Are alternative specifications of within-class heterogeneity considered (e.g., LGCA vs. LGMM) and clearly documented? If not, was sufficient justification provided as to eliminate certain specifications from consideration?  6b. Are alternative specifications of the between-class differences in variance–covariance matrix structure considered and clearly documented? If  not, was sufficient justification provided as to eliminate certain specifications from consideration? | Y  Y | The residual variances were held equal across classes to simplify the model. |
| 7. Are alternative shape/functional forms of the trajectories described? | N | The linear trajectory is more appropriate for the outcome used (gait speed) than other trajectory shapes based on a priori knowledge. |
| 8. If covariates have been used, can analyses still be replicated? | N | No covariates were used. |
| 9. Is information reported about the number of random start values and final iterations included? | N | Maximum likelihood was not used. |
| 10. Are the model comparison (and selection) tools described from a statistical perspective? | Y |  |
| 11. Are the total number of fitted models reported, including a one-class solution? | Y |  |
| 12. Are the number of cases per class reported for each model (absolute sample size, or proportion)? | Y |  |
| 13. If classification of cases in a trajectory is the goal, is entropy reported? | Y |  |
| 14a. Is a plot included with the estimated mean trajectories of the final solution?  14b. Are plots included with the estimated mean trajectories for each model?  14c. Is a plot included of the combination of estimated means of the final model and the observed individual trajectories split out for each latent class? | Y  N  Y | All arguments used to decide on the number of classes are presented in Table 2. |
| 15. Are characteristics of the final class solution numerically described (i.e., means, SD/SE, n, CI, etc.)? | Y |  |
| 16. Are the syntax files available (either in the appendix, supplementary materials, or from the authors)? | Y | Syntax files are available on request. |

LGCA = latent class growth analysis; LGMM = latent growth mixture modeling.

Supplementary Table 2. Baseline characteristics of participants in each trajectory subgroup

| variables | Good gait speed group (n=1043) | Slow gait speed group (n=246) | | *P* |
| --- | --- | --- | --- | --- |
| Age，year |  |  | <0.001 | |
| <60 | 505 (48.42) | 75 (30.49) |  | |
| ≥60 | 538 (51.58) | 171 (69.51) | |  |
| Gender |  |  | | <0.001 |
| Female | 545 (52.25) | 182 (73.98) | |  |
| Male | 498 (47.75) | 64 (26.02) | |  |
| Race |  |  | | <0.001 |
| Other | 258 (24.74) | 123 (50.00) | |  |
| White or Caucasian | 785 (75.26) | 123 (50.00) | |  |
| Marital status |  |  | | <0.001 |
| Other | 334 (32.02) | 131 (53.25) | |  |
| Married | 709 (67.98) | 115 (46.75) | |  |
| Education |  |  | | <0.001 |
| None/Primary | 179 (17.17) | 81 (32.93) | |  |
| Secondary | 506 (48.51) | 105 (42.68) | |  |
| Tertiary | 358 (34.32) | 60 (24.39) | |  |
| Income, $ |  |  | | <0.001 |
| <50,000 | 389 (37.30) | 158 (64.23) | |  |
| ≥50,000 | 654 (62.70) | 88 (35.77) | |  |
| Smoking history |  |  | | 0.413 |
| No | 471 (45.16) | 104 (42.28) | |  |
| Yes | 572 (54.84) | 142 (57.72) | |  |
| Drinking history |  |  | | <0.001 |
| No | 595 (57.05) | 181 (73.58) | |  |
| Yes | 448 (42.95) | 65 (26.42) | |  |
| Obesity |  |  | | <0.001 |
| No | 576 (55.23) | 83 (33.74) | |  |
| Yes | 467 (44.77) | 163 (66.26) | |  |
| Depressive symptoms |  |  | |  |
| No | 927 (88.88) | 186 (75.61) | | <0.001 |
| Yes | 116 (11.12) | 60 (24.39) | |  |
| Comorbidity |  |  | |  |
| No | 775 (74.30) | 144 (58.54) | | <0.001 |
| Yes | 268 (25.70) | 102 (41.46) | |  |
| History of knee injury |  |  | | 0.611 |
| No | 980 (93.96) | 229 (93.09) | |  |
| Yes | 63 (6.04) | 17 (6.91) | |  |
| History of knee surgery |  |  | | 0.048 |
| No | 646 (61.94) | 169 (68.70) | |  |
| Yes | 397 (38.06) | 77 (31.30) | |  |
| KLG |  |  | | 0.489 |
| 2 | 481 (46.12) | 104 (42.28) | |  |
| 3 | 400 (38.35) | 104 (42.28) | |  |
| 4 | 162 (15.53) | 38 (15.44) | |  |
| WOMAC pain |  |  | | <0.001 |
| <5 | 523 (50.14) | 66 (26.83) | |  |
| ≥5 | 520 (49.86) | 180 (73.17) | |  |

Obesity was defined as BMI ≥30 kg/m^2^, Depressive symptoms were defined as CES-D (catchment-area epidemiology survey-depression) score ≥16, KLG Kellgren-Lawrence grade, WOMAC Western Ontario & McMaster Universities Osteoarthritis Index.


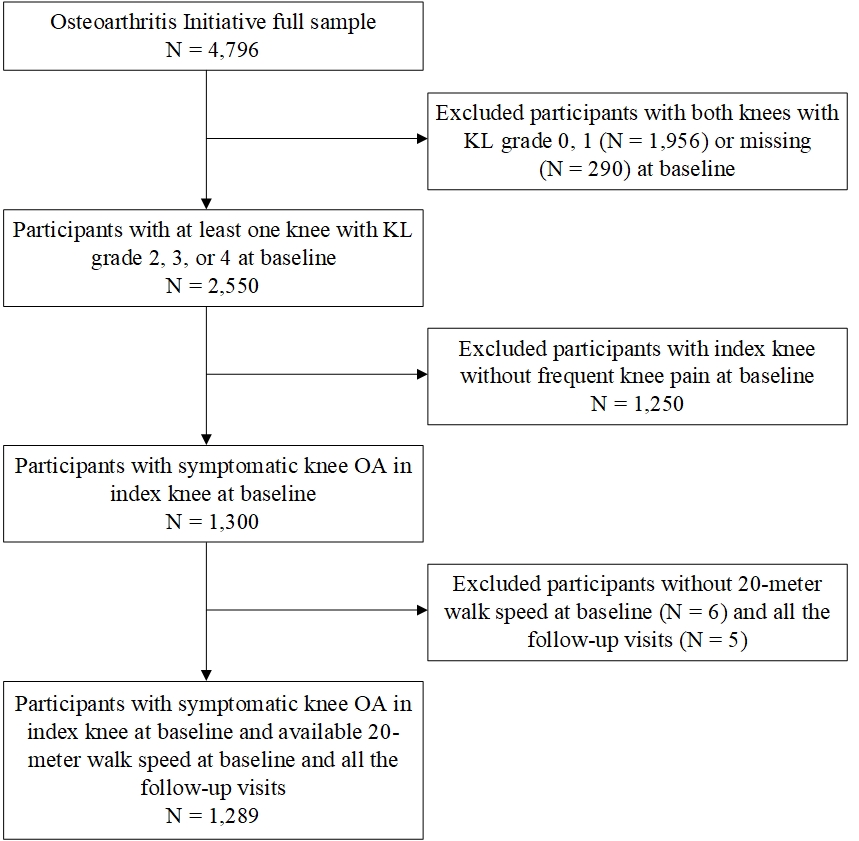


Supplementary Fig. 1 Study sample flow diagram. K-L: Kellgren-Lawrence; OA: Osteoarthritis.

Supplementary Fig. 2 Logical framework of the study.
